# Seasonality and effects of climatic exposures on community-acquired Legionnaires’ disease incidence, Italy, 2005 to 2023

**DOI:** 10.2807/1560-7917.ES.2026.31.12.2500712

**Published:** 2026-03-26

**Authors:** Antonio Sciurti, Jessica Iera, Andrea Cannone, Alberto Mateo-Urdiales, Luigi De Angelis, Valentina Baccolini, Roberta Urciuoli, Stefania Giannitelli, Giulia Fadda, Benedetta Bellini, Fabiola Mancini, Maria Scaturro, Maria Cristina Rota, Maria Luisa Ricci, Antonino Bella, Patrizio Pezzotti

**Affiliations:** 1 Department of Infectious Diseases, Istituto Superiore di Sanità, Rome, Italy; 2 Department of Public Health and Infectious Diseases, Sapienza University of Rome, Rome, Italy; 3 Department of Translational Research and New Technologies in Medicine and Surgery, University of Pisa, Pisa, Italy; 4 ESCMID Study Group for *Legionella* Infections (ESGLI), Basel, Switzerland

**Keywords:** Legionella, Legionnaires’ disease, surveillance, case time-series, climate, temperature, rainfall, humidity

## Abstract

**BACKGROUND:**

Community-acquired Legionnaires’ disease (LD) has increased globally, including Europe. Climatic exposures may contribute to this rise, yet evidence from high-incidence countries, such as Italy, is limited.

**AIM:**

We aimed to assess LD seasonality at the national level and the effects of temperature, relative humidity and precipitation on LD incidence at the municipality level.

**METHODS:**

We analysed data on notified community-acquired LD cases and national climatic data from 2005 to 2023. Seasonality was assessed (peak-to-trough ratio (PTR)). A case time-series design with distributed lag nonlinear models was applied to estimate 1–10 week lagged effects of climatic exposures on LD incidence at the municipality level, as incidence rate ratios (IRRs).

**RESULTS:**

Of the 28,662 notified LD cases, 27,458 (95.8%) were included. A clear seasonality was observed in the incidence, with a trough in early spring and a peak in autumn (PTR = 3.62; 95% confidence interval (CI): 2.45–5.34). Higher temperature and relative humidity had the strongest effects 10–9 weeks before disease onset (for 5°C increase above 15°C: IRR = 1.45; 95% CI: 1.33–1.58 and for 5% increase above 60%: IRR = 1.19; 95% CI: 1.12–1.26). Precipitation showed more immediate effects, particularly with a 1-week lag (for 5 mm increase above 10 mm: IRR = 1.07; 95% CI: 1.06–1.09). Findings were consistent among individuals aged ≥ 60 years.

**CONCLUSION:**

A clear seasonal pattern was seen in the LD incidence, peaking in summer and autumn. Temperature and humidity increased LD risk over longer lags, while precipitation had shorter-term effects, suggesting climatic exposures influence environmental proliferation and immediate transmission of *Legionella*.

**Key public health message**

**What did you want to address in this study and why?**

Legionnaires’ disease is a severe pneumonia caused by *Legionella* bacteria. People can get the infection by inhaling tiny water droplets that contain *Legionella* bacteria. These bacteria are common in the environment and live naturally in water sources. Italy reports one of the highest numbers of Legionnaires’ disease cases in Europe. We aimed to investigate how season, temperature, humidity and rainfall may affect the risk of getting the disease.

**What have we learnt from this study?**

Legionnaires’ disease cases were more common in summer and autumn. Warm and humid weather increased the risk up to 10 weeks before symptom onset, while rainfall seemed to have a quicker effect, with cases rising shortly after 1 week. This suggests that weather affects *Legionella* bacteria in more than one way.

**What are the implications of your findings for public health?**

Knowing when the risk is higher can help health services stay alert at the right time of year. It can guide doctors to test for Legionnaires’ disease sooner in certain periods, and public health professionals to monitor potential sources of infection. This can help protect people who are more vulnerable to severe illness.

## Introduction

Legionnaires’ disease (LD) is a severe pneumonia caused by Gram-negative bacteria of the genus *Legionella,* with *Legionella pneumophila* being the most common. Infection occurs when individuals inhale aerosolised droplets containing bacteria, typically released from a contaminated environmental source [1]. In the last decade, the number of reported cases and the incidence of LD have increased globally [2], including the United States (US) [3] and most European countries [4]. Most diagnosed cases are considered community-acquired and sporadic, as they cannot be linked to any known or confirmed source of exposure [4,5].

Several factors may be contributing to the increase, including an ageing population, resulting in older adults vulnerable to *Legionella* infection. Additionally, improved diagnostic methods and surveillance systems with increased sensitivity may have led to increased case identification [6]. Furthermore, climatic factors may also play a role in the increase. A recent systematic review highlighted the need for more evidence on the relationship between climatic factors and LD and emphasised that studies from European countries with a high LD incidence are still lacking [7].

In 2021, Italy was one of the countries with the highest number of notified LD cases and incidence in Europe, according to the latest report on LD from the European Centre for Disease Prevention and Control (ECDC) [4]. A rising trend in incidence is further confirmed by the latest national Italian surveillance report [8]. To date, no comprehensive evaluation has been conducted on the seasonality and impact of climatic exposures on LD incidence in Italy. Therefore, we aimed to assess the seasonality of LD at the national level, as well as the effects of climatic exposures, specifically temperature, relative humidity and precipitation, at the municipality level, using Italian surveillance data from 2005 to 2023.

## Methods

### Surveillance of Legionnaires’ disease in Italy

Legionnaires’ disease is mandatorily notifiable in Italy, and surveillance is coordinated by the Department of Infectious Diseases of the Italian National Institute of Health (Istituto Superiore di Sanità, ISS) since 1990 [9]. The surveillance system follows the European Union (EU) definition for confirmed and probable LD cases [10]. Briefly, a confirmed case of LD is a patient with pneumonia and with definitive laboratory evidence of *Legionella* infection, such as isolation of the bacteria from respiratory secretions, detection of *L. pneumophila* urinary antigen, or a specific antibody response to *L. pneumophila* serogroup 1. A probable case, instead, is a person with pneumonia and with less definitive laboratory evidence (e.g. detection of *L. pneumophila* antigen in respiratory secretions, detection of *L. pneumophila* nucleic acid, or specific antibody findings), or a person with pneumonia and with no laboratory evidence of *L. pneumophila*, but a history of exposure to a contaminated environment or a common infection source [10]. Community-acquired LD case is a patient who, in the 10 days before symptom onset, has neither been hospitalised nor stayed in an accommodation facility, such as care home, hotel or other lodging [9,11]. For each reported LD case, a set of information is collected, including age, sex, date of symptom onset, type of laboratory test (isolation of *Legionella* spp., detection of *L. pneumophila* antigen in urine, detection of *Legionella* spp. in respiratory secretions with PCR, considerable rise in specific antibody level to *L. pneumophila* in paired serum samples or high level of *L. pneumophila*-specific antibody in a single serum sample), date of hospitalisation, setting of exposure (community, travel, healthcare facility, or other), municipality of residence, municipality of stay, hospital or healthcare facility (including name and hospital code).

### Municipalities and demographic data

The Italian territory is administratively divided into 19 regions (Nomenclature of Territorial Units for Statistics level 2 (NUTS 2)) and two autonomous provinces (APs) (NUTS 3), which are further subdivided into 7,896 municipalities, the smallest territorial units. During the 19-year study period, some municipalities were abolished, renamed or aggregated. Thus, we used the administrative unit codes and municipality boundaries of January 2024, and their corresponding shapefile, as provided by the Italian National Institute of Statistics (Istituto Nazionale di Statistica, Istat; https://www.istat.it/notizia/confini-delle-unita-amministrative-a-fini-statistici-al-1-gennaio-2018-2/). The total number of residents on 1 January from 2005 to 2023 in each municipality, stratified by age, was derived from demographic data available on the Istat website (https://www.istat.it/).

### Climatic data

Climatic variables were extracted from the Very High-Resolution REAnalysis over Italy (VHR-REA IT) dataset (https://dds.cmcc.it/#/dataset/era5-downscaled-over-italy/hourly) [12]. This dataset provides dynamically downscaled raster data from the European Centre for Medium-Range Weather Forecasts (ECMF) reanalysis version 5 (ERA5), with a horizontal spatial resolution of 2.2 × 2.2 km and a temporal resolution of 1 h, covering the entire Italian territory from 1981 to 2023.

The following climatic variables were extracted from 2005 to 2023: 2 m above the ground (2 m) temperature (Kelvin (K)), 2 m dew point temperature (K) and total precipitation (kg/m^2^, corresponding approximately to mm). The 2 m temperature was converted to Celsius (°C), and relative humidity (%) was calculated from the 2 m temperature and the 2 m dew point temperature using the World Meteorological Organization (WMO) method [13]. Hourly raster data were aggregated by week as defined by the International Organization for Standardization (ISO): 2 m temperature and relative humidity were aggregated into a weekly mean, while total precipitation was aggregated into a weekly sum. Finally, we computed weekly area-weighted averages for each of the climatic variables for the Italian territory and for each municipality. In detail, the area-weighted average is the mean value of raster cells intersecting a polygon, i.e. the boundaries of Italian territory or municipalities, weighted by the fraction of the cell that is covered.

As climatic variables were available up to 2023 (as of 30 May 2025), LD surveillance and demographic data were extracted up to the same year.

### Study design and statistical analysis

For this study, only notified cases of community-acquired LD diagnosed from 2005 to 2023 were included. We chose 2005 as the first year because 20 of 21 Italian regions/APs notified at least one community-acquired LD case in this year. The annual number of notified LD cases from 1995 to 2023 is shown in Supplementary Figure S1. Moreover, microbiological legionellosis diagnosis guidelines were issued in 2005 in Italy [14]. The exposure location was considered the municipality of stay. If the municipality of stay (where the case normally lived) was not reported, the municipality of residence (where the case was registered to live) or the municipality of the hospital was used as a proxy. Finally, community-acquired LD cases with missing or unknown stay or residence and hospitalisation facility municipality were excluded. Weekly series of LD cases, both at the national and at the municipality level, were computed aggregating the cases by the ISO week of symptom onset or hospitalisation if the date of symptom onset was missing or unknown. Similarly, weekly series of area-weighted average for mean temperature, mean relative humidity and total precipitation were collected both at the national and municipality level, representing the climatic exposures of interest.

#### Assessment of seasonality at the national level

To examine the seasonality of community-acquired LD, we used a quasi-Poisson regression model for weekly LD cases with a cyclic spline function for week-of-year with 4 degrees of freedom [15] and the logarithm of the annual population as an offset. To control for long-term trends and the effect of month of the year, we included a stratum term defined by year and month of the year. In this way, we were able to fit a seasonal curve of community-acquired LD incidence and identify the timing of incidence peaks (i.e. the week-of-year with maximum incidence estimate) and incidence troughs (i.e. the week-of-year minimum incidence estimate), and compute their 95% empirical confidence intervals (eCIs) using a Monte Carlo simulation, and peak-to-trough ratio (PTR) with its 95% confidence intervals (CI), representing the seasonal curve amplitude [15]. Finally, to visually inspect the relationship between seasonality of community-acquired LD and climatic exposures, the seasonality curve was plotted and overlaid with weekly average temperature, relative humidity and total precipitation in Italy across years.

#### Assessment of climatic exposures effects at the municipality level

The case time series (CTS) design was used to examine the relationship between climatic exposures and community-acquired LD incidence at the municipality level, with municipalities the observational units. Within each municipality, weekly community-acquired LD cases were the outcome and lagged weekly area-weighted average climatic variables were the exposures of interest [16]. We excluded the week of symptom onset, i.e. lag of 0 weeks, and considered a lag of 10 to 1 weeks, according to the median incubation period of the disease (7 days) [17] and potentially longer lagged effects of environmental exposures on community-acquired LD incidence. For instance, some studies have reported temperature effects to be stronger 2–9 weeks prior to symptom onset, potentially reflecting its influence on *Legionella* spp. growth in the environment [18-21]. The CTS design was implemented using a multivariable conditional quasi-Poisson model for community-acquired LD incidence to manage overdispersion. The model included a stratum term defined by municipality, year and month of the year and incorporated the logarithm of the population as offset. By doing so, the analysis was limited to strata with at least one community-acquired LD case during the study period, allowing for assessing short-term effects by comparing time periods in proximity (e.g. within the same municipality and month) and thus capturing within-stratum risk variations. To evaluate the 10-week lagged effects of climatic exposures on LD cases, we used a distributed lag nonlinear model (DLNM). These are two-dimensional models designed to analyse the delayed and nonlinear effects of an exposure on an outcome, and are specified by defining a cross-basis, a bi-dimensional space of functions that represents at the same time the dependency along both the range of the exposure and the lag dimension of its occurrence [22]. In our analysis, climatic exposures were represented using a linear function, while the lag effect was captured through a natural spline with one to three equally spaced knots on the logarithmic scale. The multivariable model incorporated the cross-bases for all weekly area-weighted average climatic exposures, i.e. weekly mean temperature, weekly mean relative humidity and weekly total precipitation, lagged from 10 to 1 week. In this way, for each of the 10-week lags, incidence rate ratios (IRRs) and their 95% CIs were estimated at specific exposure values with a defined reference value (temperature: 0–35°C (reference: 15°C); relative humidity: 20–80% (reference: 60%); total precipitation: 0–50 mm (reference: 10 mm)). We then calculated the combined effect of all exposures within each lag, by multiplying the exposure-specific IRRs estimated from the model, using the same defined reference values. Finally, given Italy’s ageing population [23] and the higher risk of LD among older adults [24], a sensitivity analysis was conducted by restricting the population to individuals aged ≥ 60 years, to account for the potential effect of age on community-acquired LD incidence.

We used R statistical software for the analyses (version 4.2.3; R Core Team 2023, R Foundation for Statistical Computing, Vienna, Austria). The R packages *terra*, *sf*, *exactextractr*, *dlnm* and *gnm* were used.

## Results

### Descriptive analyses

**Figure 1:**
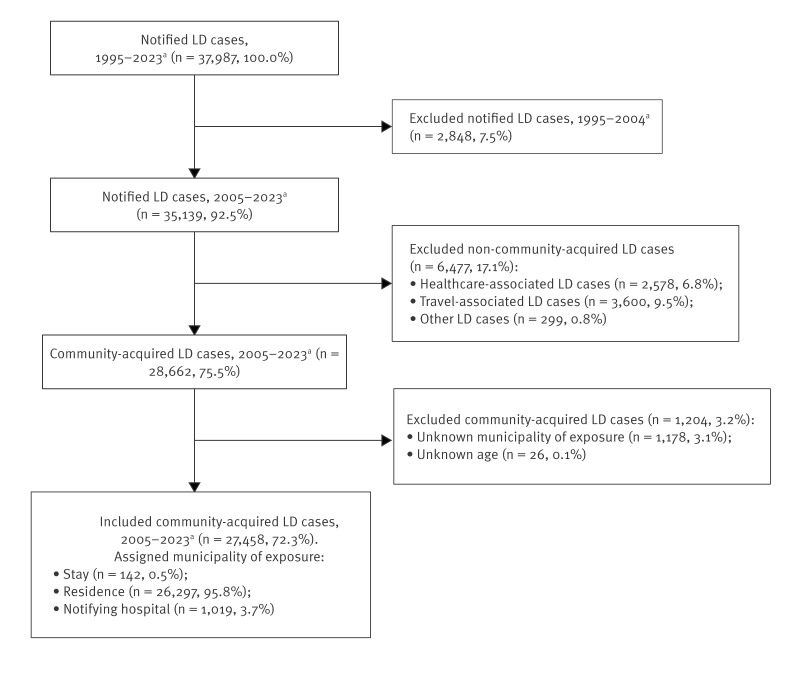
Flow chart of inclusion of community-acquired cases with Legionnaires’ disease in a study on seasonality and effects of climatic exposures on community-acquired Legionnaires’ disease incidence, Italy, 2005–2023 (n = 27,458 cases)

The inclusion of community-acquired LD cases in the study is illustrated in Figure 1. Between 2005 and 2023, 28,662 community-acquired LD cases were notified in Italy, of which 27,458 (95.8%) cases were included in the study, while 1,178 (4.1%) community-acquired LD cases were excluded due to unknown municipality of exposure and 26 (0.1%) due to unknown age. The included cases represented 72.3% of all notified cases since the start of the surveillance in 1995 (n = 37,987). The municipality of exposure was considered the residence municipality in 95.8% of cases (n = 26,297), followed by the municipality of the notifying hospital (3.7%, n = 1,019) and municipality of stay (0.5%, n = 142).

LD: Legionnaires’ disease.

^a^ Year of symptom onset.

Percentages in the figure are calculated from the total number of LD cases 1995–2023.

**Figure 2:**
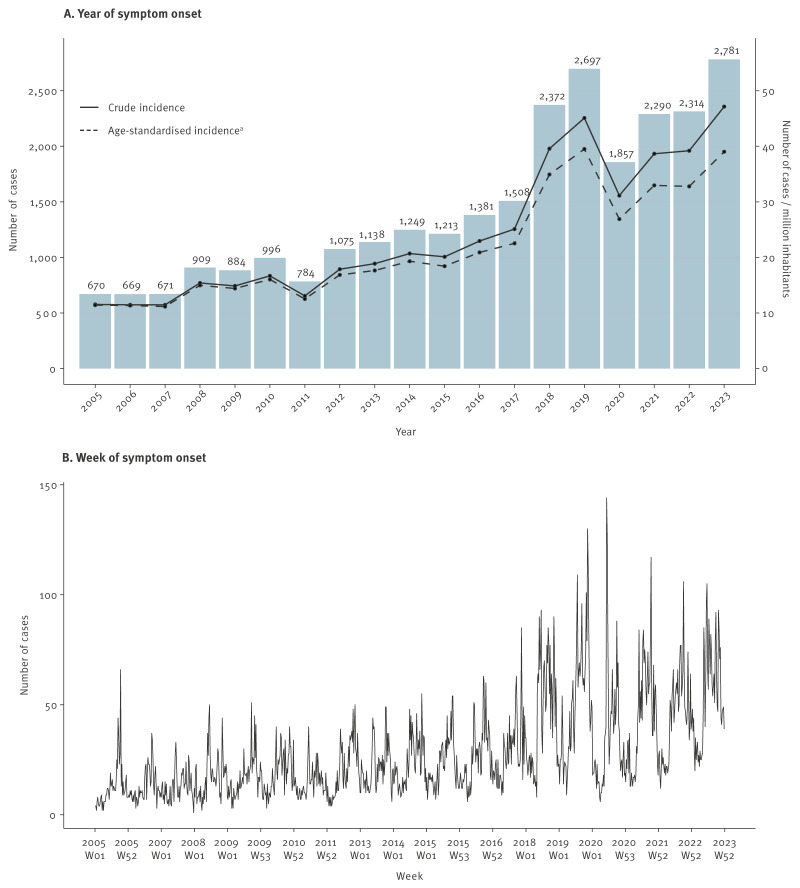
Number and incidence (per 1 million inhabitants) of notified community-acquired cases with Legionnaires’ disease, by year and week of symptom onset, Italy, 2005–2023 (n = 27,458 cases)

^a^ Direct standardisation, 19 age groups up to > 90 years. Reference population was the European Standard Population [50].

The median age of cases was 66 years (interquartile range (IQR): 54.0–77.0 years), 71.2% were males (n = 19,538) and 28.8% were females (n = 7,920). Most cases were confirmed by detection of *L. pneumophila* antigen in urine (95.3%, n = 26,162). The remaining were identified by high levels of *L. pneumophila*-specific antibody in a single sample (1.8%, n = 481), multiple tests (1.1%, n = 297), isolation (1.0%, n = 266) and detection of *Legionella* spp. in respiratory samples with PCR (0.9%, n = 252). An increase in the number of notified community-acquired LD cases in Italy was observed from 2005 to 2023 (Figure 2A). The annual incidence followed a similar pattern, with 47.1 cases per 1 million inhabitants in 2023 and the age-standardised incidence slightly lower (Figure 2A).

Panel A. The COVID-19 pandemic started in 2020.

### Seasonality at the national level

The weekly number of cases increased over the study period, displaying an apparent seasonal pattern, with troughs in spring and peaks in summer and autumn (Figure 2B). A similar trend was observed for weekly average temperatures, with peaks in summer season and troughs in winter. In contrast, relative humidity exhibited an opposite trend, peaking in the colder months and dipping in summer. Precipitation levels showed greater variability, with less consistent seasonal pattern. Weekly trends of community-acquired LD cases and temperature, relative humidity and total precipitation are shown in Supplementary Figure S2.

A decreasing median proportion of community-acquired LD cases across years was found from January to April (range: 3.8–5.2%), followed by a rise peaking in June (10.2%) and September–October (12.0%). The fitted seasonality curve confirmed this pattern, with an estimated incidence trough in week 10 (95% eCI: week 8–12), peak in week 40 (95% eCI: week 26–43) and a PTR of 3.62 (95% CI: 2.45–5.34). We present the median proportion and IQR of community-acquired LD cases across years and the fitted seasonality curve in Supplementary Figure S3.

**Figure 3:**
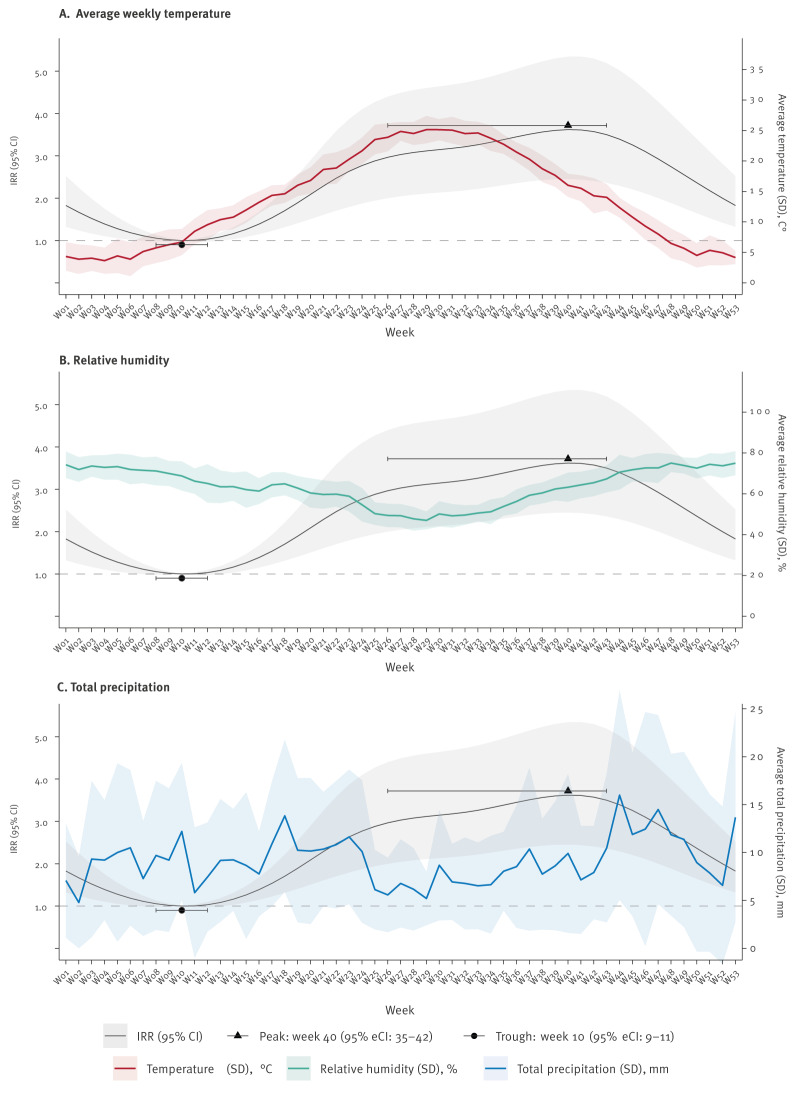
Seasonality of community-acquired cases with Legionnaires’ disease, by temperature, humidity and precipitation, Italy, 2005–2023 (n = 27,458 cases)

CI: confidence interval; eCI: empirical confidence interval; IRR: incidence rate ratio; LD: Legionnaires’ disease; SD: standard deviation.

Average weekly temperature peaked in week 29 (25.1 ± 2.2°C) (Figure 3A), and average relative humidity had an inverse trend, reaching a trough in week 29 (47.0 ± 4.3%) (Figure 3B). A similar, but less evident pattern was observed for average total precipitation, with lower values approximately between week 25 and 34 (Figure 3C).

IRR is the ratio of LD incidence in each week-of-year to LD incidence in the trough week.

### Climatic exposures effects at the municipality level

**Figure 4:**
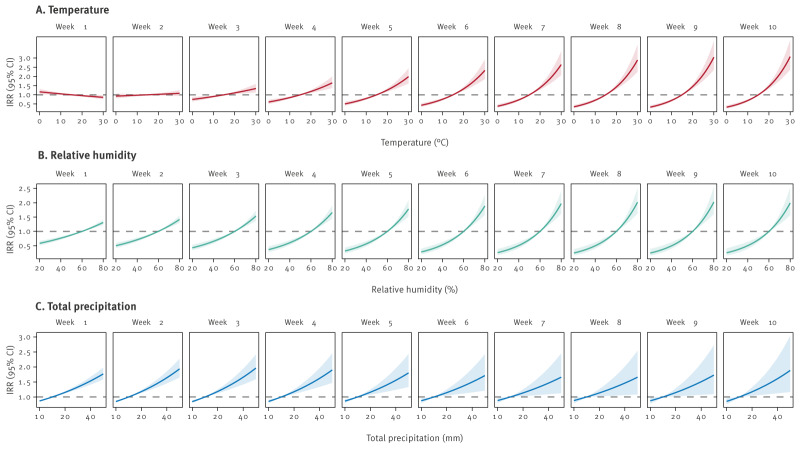
The effect of temperature, relative humidity and total precipitation on the incidence of community-acquired Legionnaires’ disease at the municipality level, by weekly lag, Italy, 2005–2023 (n  = 27,458 cases)^a^

CI: confidence interval; IRR: incidence rate ratio; LD: Legionnaires’ disease.

^a^ 4,107 municipalities included.

In 4,107 (52.0%) of the 7,896 municipalities, at least one community-acquired LD case was identified and included in the analysis. From 2005 to 2023, the overall yearly population in these 4,107 municipalities was 50,748,116 inhabitants on average (range: 49,221,215–51,456,140), while the remaining 3,789 municipalities without cases had altogether an average of 8,768,678 inhabitants (range: 8,410,347–8,949,548). Figure 4 illustrates the weekly lag-specific effects of climatic exposures on community-acquired LD incidence at the municipality level, as estimated by the multivariable model. The corresponding IRRs and 95% CIs are shown in Supplementary Table S1. From lags of 10 to 3 weeks, temperatures above 15°C were associated with a higher incidence (5°C increase above 15°C: from IRR = 1.45 (95% CI: 1.33–1.58) with 10-week lags to IRR = 1.10 (95% CI: 1.04–1.17) with 3-week lags), while temperatures below 15°C were associated with reduced incidence (5°C decrease below 15°C: from IRR = 0.91 (95% CI: 0.86–0.96) with 10-week lags to IRR = 0.69 (95% CI: 0.63–0.75) with 3-week lags) (Figure 4A). With 2-week lags, the temperature effect was negligible (5°C increase above 15°C: IRR = 1.02; 95% CI: 0.98–1.08), and it slightly reversed direction with 1-week lags (5°C increase above 15°C: IRR = 0.95; 95% CI: 0.91–0.99). Relative humidity showed a similar pattern: values above 60% associated with higher incidence (5% increase above 60%: from IRR = 1.19 (95% CI: 1.12–1.26) with 10-week lags to IRR = 1.07 (95% CI: 1.05–1.09) with 1-week lags), and values below 60% with lower incidence across all week lags (5% decrease below 60%: from IRR = 0.84 (95% CI: 0.79–0.89) with 10-week lags to IRR = 0.93 (95% CI: 0.92–0.95) with 1-week lags) (Figure 4B). For total precipitation (Figure 4C), rainfall above 10 mm was associated with higher incidence (5 mm increase above 10 mm: from IRR = 1.08 (95% CI: 1.04–1.12) with 5-week lags to IRR = 1.07 (95% CI: 1.06–1.09) with 1-week lags; and from IRR = 1.08 (95% CI: 1.02–1.15) with 10-week lags to IRR = 1.07 (95% CI: 1.02–1.12) with 6-week lags). Conversely, precipitation below 10 mm was associated with lower incidence (5 mm decrease below 10 mm: from IRR = 0.92 (95% CI: 0.87–0.98) with 10-week lags to IRR = 0.93 (95% CI: 0.92–0.94) with 1-week lags).

IRRs and 95% CIs were estimated with a multivariable conditional quasi-Poisson model for community-acquired LD incidence at the municipality level. The model included three cross-bases, one for each area-weighted average climatic exposure (weekly mean temperature, weekly mean relative humidity and weekly total precipitation), with lags from 1 to 10 week. IRRs and 95% CIs are reported for individual week lags.

**Figure 5:**
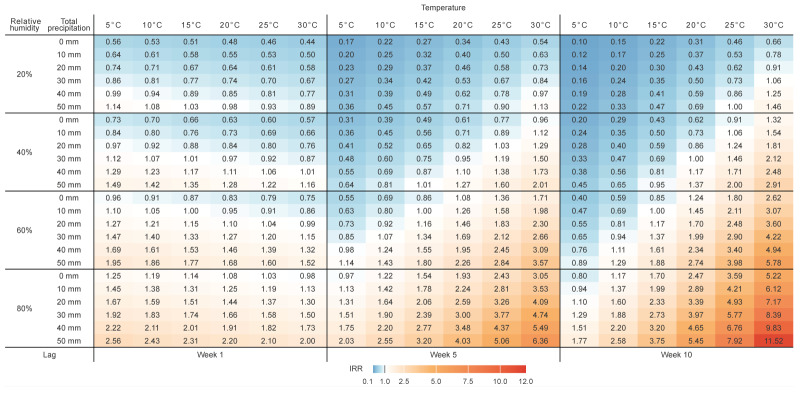
Combined effect of temperature, relative humidity and total precipitation on the incidence of community-acquired LD Legionnaires’ disease at the municipality level, by week, Italy, 2005–2023 (n = 27,458 cases)^a^

IRR: incidence rate ratio; LD: Legionnaires’ disease.

^a^ 4,107 municipalities included.

Incidence rate ratios combined for all the exposures with lags of 10, 5 and 1 week are presented in Figure 5. Compared with reference exposure values (i.e. 15°C temperature, 60% humidity and 10 mm total precipitation), higher levels of temperature, relative humidity and total precipitation were associated with increased incidence with both 10-week lags and, to a lesser extent, 5-week lags. In contrast, with 1-week lags, higher temperatures were associated with lower incidence, although these associations were weaker than those observed with lags of 10 and 5 weeks.

Combined IRRs were calculated by multiplying the exposure-specific IRRs estimated from the multivariable conditional quasi-Poisson model for community-acquired LD incidence at the municipality level. The model included three cross-bases, one for each area-weighted average climatic exposure (weekly mean temperature, weekly mean relative humidity and weekly total precipitation), with lags from 1 to 10 weeks. Combined IRRs are reported for individual week lags 1, 5 and 10.

The sensitivity analysis conducted in the population of individuals aged ≥ 60 years yielded similar results. This analysis is shown in Supplementary Figures S4 and S5.

## Discussion

We investigated the seasonality of community-acquired LD and the effect of climatic exposures on the incidence of LD in Italy. We found a clear seasonal pattern, with incidence peaking in late summer and autumn and declining in winter and spring. Similar summer peaks have been reported in other European countries [25], the US [3] and China [26].

Regarding climatic factors at the municipality level, both higher mean weekly temperature and relative humidity in earlier weeks appeared to increase LD incidence, with the strongest effects observed 10–5 weeks before occurrence, whereas precipitation showed a more apparent positive effect in weeks immediately preceding LD occurrence. Temperature displayed a slightly negative association 1 week before onset of symptoms of LD. Comparable results emerged when restricting analyses to adults aged ≥ 60 years.

Few studies have measured climatic effects over long lags. Some reported increased risk of LD with higher temperature 2–4 weeks [20,21] or over 9 weeks [18,19] before symptom onset. Most research, however, has focused on shorter lags (0–20 days) [19,21,27-31], consistent with the time between exposure and symptom onset of LD, and found that precipitation and/or humidity had greater short-term effects than temperature [19,27,29-32]. In the days before onset, temperature was often either not associated [19,27,29,30] or inversely associated with LD [21,28]. Our results are consistent, suggesting that hot and humid weather may promote LD risk over longer lags, up to 10 weeks, whereas rainy weather appears to increase in weeks closer to LD occurrence, and hot and dry conditions may be protective in the week immediately before symptom onset.

Climatic exposures may influence LD risk by enhancing proliferation in the environment, promoting transmission, or both. The observed 10–5 week lags for temperature and humidity effects may reflect the growth of *Legionella* spp., its thermophilic hosts such as free-living amoebae [33] and other supportive microorganisms [34]. Under laboratory conditions, numbers of *L. pneumophila* cells peaked in tap water after 4 weeks at 37°C [35], but growth in non-experimental settings may take longer. Precipitation may facilitate transmission over shorter periods by dispersing aerosols from contaminated sources [36] or by disrupting public water supply quality [31]. Similarly, humid weather may prolong *Legionella* survival in aerosols [37], whereas hot and dry conditions may reduce their viability [38]. However, despite biological plausibility, additional evidence linking higher *Legionella* concentrations and viability in environmental sources, both artificial (e.g. cooling towers, fountains) and natural (e.g. ponds, lakes, soil), to warmer and more humid periods, is required to establish a clearer preventive understanding of the observed climatic associations. These future findings may also contribute to clarifying investigations of travel-associated cases.

The extension of the CTS design to small areas reduces ecological bias by aligning the measurement of exposures and outcomes, and offering better control for confounding [16], including behavioural factors that follow a seasonal pattern and could independently influence exposure to environmental *Legionella* sources (e.g. outdoor activities in warmer months). To ascertain municipal-level climatic exposures over a 19-year period, we used climate re-analysis data, as they offer more consistent long-term coverage and higher spatial resolution than conventional weather station records [39].

Some limitations should be acknowledged. Firstly, LD surveillance data were not collected for this study’s specific purpose, and exposure location was assumed to be the municipality of residence for 96% of community-acquired LD cases, with 4% assigned to the municipality of the notifying hospital. This may not always reflect the true exposure site (e.g. for cases with long-term stays or hospitalisation outside their municipality of residence). In addition, cases were grouped by week of symptom onset (lag 0), assuming a median 7-day incubation period [17]. Thus, the exposures of cases with shorter or longer incubation periods may have been misclassified. Cases with an incubation period shorter than 1 week could have been infected during week 0, whereas those with longer incubation periods might not have been affected by exposures in week 1. Ideally, if the time point for transmission were known, exposures during week 0 should be included for cases with shorter incubation periods and weeks 0 and 1 should be excluded for cases with longer incubation periods. However, we consider the week of symptom onset a reliable proxy for LD, since some initial LD symptoms are nonspecific (e.g. headache, fatigue) and patients may not always recall the exact date their symptoms began. In addition, underdiagnosis and underreporting, particularly for mild or non-hospitalised LD cases, are possible [40]. Only about half of the municipalities were included in the study, which may further raise concerns about underdiagnosis and underreporting. However, the included municipalities accounted for roughly 50.7 million people, whereas those without any cases accounted for ca 8.8 million, indicating that most of the at-risk population was still represented in the analysis. For climatic exposures, we used weekly municipal area-weighted averages for mean temperature, mean relative humidity and total precipitation. This approach may have introduced some bias towards the null, potentially underestimating the observed associations and suggesting that the true effect sizes may be larger than our estimates indicate. Also, since exposure to temperature, relative humidity and precipitation likely varies within municipalities, particularly across municipalities with areas at different elevation (e.g. mountainous, hilly, flat), the use of area-weighted average exposures likely diluted these potential differences. Other factors, such as geographical region, elevation, population density, urbanisation degree or status of water supply network may influence the occurrence of community-acquired LD cases at the municipality level, but as these factors do not vary meaningfully over time within each stratum, the CTS framework inherently controls for these potential time-invariant confounders [16]. However, future studies examining time-invariant characteristics of municipalities, such as degree of urbanisation and water supply system status, may help clarify their role in the occurrence of community-acquired LD cases. Moreover, since urinary antigen testing does not reliably detect *Legionella* species and serogroups other than *L. pneumophila* serogroup 1 [41], and most community-acquired LD cases (95.3%) were diagnosed using that method, the climatic associations identified are mainly driven by *L. pneumophila* serogroup 1 infections. It should be noted, however, that *L. pneumophila* serogroup 1 is the most frequently identified serogroup both in culture-confirmed cases and samples taken from artificial environmental sources [4,8], and has a greater potential for dissemination in the environment and infection, possibly due to its unique surface lipopolysaccharide conformation and hydrophobic characteristics [42]. By contrast, other *L. pneumophila* serogroups or non-*pneumophila Legionella* species may be found in various environmental sources and have different sensitivity to climatic conditions.

Finally, not all drivers of Italy’s LD increase were addressed. We identified climatic exposures as potential contributors to this rise – and climate change, characterised by rising temperatures and more frequent extreme precipitation events, as observed in Italy and worldwide [43,44], is likely to have played a relevant role [45]. However, other factors, such as population ageing and improved diagnosis and surveillance, are also likely to contribute. Ageing was associated with increased incidence over time, as crude rates rose less than age-standardised rates. Improved access to diagnostic tools, such as urinary antigen testing [5,26] and PCR-based methods [6,46], have likely contributed to enhance case detection, but no other major changes in diagnostic activity were reported at the European level [25]. Updates to Italian prevention guidelines in 2005 and 2015 [9,14] may have raised health practitioner awareness, potentially influencing case detection. However, future studies are required to explore trends and seasonal variations of diagnostic practices for community-acquired pneumonia. In this context, the decline in community-acquired LD incidence observed in 2020 may reflect the role of both behavioural and societal factors induced by restrictive measures to control severe acute respiratory syndrome coronavirus 2 transmission (e.g. lockdowns), as well as reduced diagnostic activity for *Legionella* infections in favour of COVID-19 testing [47,48]. In addition, accumulating damage to public water supply systems across the years could also be responsible for the rise in community-acquired LD case [49]. Further research is needed to quantify the contributions of these factors.

## Conclusion

In Italy, community-acquired LD shows a marked seasonal pattern, with incidence peaking in late summer and autumn. Warmer temperatures and higher humidity appear to increase LD risk several weeks before onset, while precipitation has a more immediate effect. These findings suggest that climatic conditions play an important role in shaping the timing and extent of LD occurrence. From a public health perspective, recognising high-incidence periods may prompt earlier clinical suspicion and testing of LD in pneumonia cases, while identifying climatic risk conditions may support more timely monitoring and control of potential environmental sources and inform tailored recommendations for at-risk people, such as older adults, for example, advising them to limit outdoor activities during rainfall to reduce exposure risk. Anticipating these risk periods could help strengthen prevention strategies and protect vulnerable groups, particularly in the context of ongoing climate change.

## Data Availability

The individual-level data analysed in this study cannot be publicly shared due to privacy restrictions. Aggregated data are available on a reasonable case-by-case request to the Istituto Superiore di Sanità.

## Supplementary Data

524000 Supplementary Material
