# Supplementary material for: Seasonality and effects of climatic exposures on community-acquired Legionnaires’ disease incidence, Italy, 2005 to 2023
Source: Euro Surveill. 2026 Mar 26;31(12):2500712. doi: 10.2807/1560-7917.ES.2026.31.12.2500712 (PMC13074369; doi:10.2807/1560-7917.ES.2026.31.12.2500712)
Supplement: Supplementary Material [file 25-00712_SCIURTI_Supplement.pdf]

This supplementary material is hosted by *Eurosurveillance* as supporting information alongside the article *Seasonality and effects of climatic exposures on community-acquired Legionnaires' Disease incidence: an evaluation of the Italian surveillance data, 2005-2023*, on behalf of the authors, who remain responsible for the accuracy and appropriateness of the content. The same standards for ethics, copyright, attributions and permissions as for the article apply. Supplements are not edited by *Eurosurveillance* and the journal is not responsible for the maintenance of any links or email addresses provided therein.

Table of Contents

Supplementary Table S1, page 3
Supplementary Figure S1, page 4
Supplementary Figure S2, page 5
Supplementary Figure S3, page 6
Supplementary Figure S4, page 7
Supplementary Figure S5, page 8

Supplementary Table S1. Week lag-specific IRRs and 95% CIs for effect of temperature (ref. 15 °C), relative humidity (ref. 60%) and total precipitation (ref. 10 mm) on community-acquired LD incidence at the municipality level (n. municipalities = 4107; n. community-acquired LD cases = 27,458)

Climatic exposure	Value	Week 1	Week 2	Week 3	Week 4	Week 5	Week 6	Week 7	Week 8	Week 9	Week 10
	0 °C	1.16 (1.03–1.31)	0.93 (0.80–1.07)	0.75 (0.63–0.88)	0.61 (0.50–0.73)	0.50 (0.41–0.62)	0.43 (0.34–0.54)	0.38 (0.30–0.48)	0.35 (0.27–0.44)	0.33 (0.26–0.42)	0.33 (0.25–0.42)
	5 °C	1.10 (1.02–1.20)	0.95 (0.87–1.05)	0.82 (0.73–0.92)	0.72 (0.63–0.81)	0.63 (0.55–0.73)	0.57 (0.49–0.66)	0.52 (0.45–0.62)	0.49 (0.42–0.58)	0.48 (0.40–0.56)	0.47 (0.40–0.56)
	10 °C	1.05 (1.01–1.09)	0.98 (0.93–1.02)	0.91 (0.86–0.96)	0.85 (0.80–0.90)	0.80 (0.74–0.85)	0.75 (0.70–0.81)	0.72 (0.67–0.78)	0.70 (0.65–0.76)	0.69 (0.63–0.75)	0.69 (0.63–0.75)
Temperature	15 °C	Ref.	Ref.	Ref.	Ref.	Ref.	Ref.	Ref.	Ref.	Ref.	Ref.
	20 °C	0.95 (0.91–0.99)	1.02 (0.98–1.08)	1.10 (1.04–1.17)	1.18 (1.11–1.26)	1.26 (1.17–1.35)	1.33 (1.23–1.43)	1.38 (1.28–1.50)	1.42 (1.31–1.55)	1.45 (1.33–1.58)	1.45 (1.33–1.58)
	25 °C	0.91 (0.83–0.98)	1.05 (0.95–1.16)	1.22 (1.09–1.36)	1.40 (1.23–1.58)	1.58 (1.38–1.81)	1.76 (1.51–2.04)	1.91 (1.63–2.24)	2.03 (1.72–2.40)	2.10 (1.77–2.48)	2.11 (1.78–2.51)
	30 °C	0.86 (0.76–0.97)	1.08 (0.93–1.24)	1.34 (1.13–1.59)	1.65 (1.36–1.99)	1.98 (1.61–2.44)	2.33 (1.86–2.92)	2.64 (2.07–3.36)	2.89 (2.25–3.71)	3.04 (2.36–3.92)	3.07 (2.37–3.98)
	20%	0.58 (0.51–0.67)	0.50 (0.42–0.60)	0.43 (0.34–0.53)	0.36 (0.28–0.48)	0.32 (0.23–0.43)	0.28 (0.20–0.40)	0.26 (0.17–0.38)	0.25 (0.16–0.38)	0.24 (0.15–0.39)	0.25 (0.16–0.41)
	25%	0.62 (0.55–0.70)	0.54 (0.47–0.64)	0.47 (0.39–0.58)	0.41 (0.32–0.52)	0.36 (0.28–0.48)	0.33 (0.24–0.45)	0.31 (0.22–0.43)	0.29 (0.20–0.43)	0.29 (0.19–0.43)	0.30 (0.20–0.46)
	30%	0.67 (0.60–0.74)	0.59 (0.52–0.68)	0.53 (0.44–0.62)	0.47 (0.38–0.57)	0.42 (0.33–0.53)	0.39 (0.29–0.51)	0.36 (0.27–0.49)	0.35 (0.25–0.48)	0.35 (0.25–0.49)	0.36 (0.25–0.51)
	35%	0.71 (0.66–0.78)	0.65 (0.58–0.73)	0.59 (0.51–0.68)	0.53 (0.45–0.63)	0.49 (0.40–0.59)	0.45 (0.36–0.57)	0.43 (0.33–0.55)	0.42 (0.32–0.55)	0.41 (0.31–0.55)	0.42 (0.31–0.57)
	40%	0.76 (0.71–0.82)	0.71 (0.65–0.77)	0.65 (0.58–0.73)	0.60 (0.53–0.69)	0.56 (0.48–0.66)	0.53 (0.44–0.63)	0.51 (0.42–0.62)	0.50 (0.40–0.62)	0.49 (0.39–0.62)	0.50 (0.39–0.64)
	45%	0.82 (0.78–0.86)	0.77 (0.72–0.82)	0.73 (0.67–0.79)	0.68 (0.62–0.76)	0.65 (0.58–0.73)	0.62 (0.54–0.71)	0.60 (0.52–0.70)	0.59 (0.50–0.70)	0.59 (0.50–0.70)	0.60 (0.50–0.71)
Relative humidity	50%	0.87 (0.84–0.90)	0.84 (0.80–0.88)	0.81 (0.76–0.85)	0.78 (0.73–0.83)	0.75 (0.69–0.81)	0.73 (0.67–0.80)	0.71 (0.64–0.79)	0.70 (0.63–0.78)	0.70 (0.63–0.79)	0.71 (0.63–0.80)
	55%	0.93 (0.92–0.95)	0.92 (0.90–0.94)	0.90 (0.87–0.92)	0.88 (0.85–0.91)	0.87 (0.83–0.90)	0.85 (0.82–0.89)	0.84 (0.80–0.89)	0.84 (0.79–0.89)	0.84 (0.79–0.89)	0.84 (0.79–0.89)
	60%	Ref.	Ref.	Ref.	Ref.	Ref.	Ref.	Ref.	Ref.	Ref.	Ref.
	65%	1.07 (1.05–1.09)	1.09 (1.07–1.12)	1.11 (1.08–1.15)	1.13 (1.10–1.17)	1.16 (1.11–1.20)	1.17 (1.12–1.23)	1.18 (1.13–1.25)	1.19 (1.13–1.26)	1.19 (1.13–1.26)	1.19 (1.12–1.26)
	70%	1.14 (1.11–1.18)	1.19 (1.14–1.24)	1.24 (1.17–1.31)	1.29 (1.20–1.38)	1.33 (1.23–1.44)	1.37 (1.26–1.50)	1.40 (1.27–1.55)	1.42 (1.27–1.58)	1.42 (1.27–1.60)	1.41 (1.25–1.59)
	75%	1.22 (1.16–1.29)	1.30 (1.21–1.39)	1.38 (1.26–1.50)	1.46 (1.32–1.62)	1.54 (1.37–1.74)	1.61 (1.41–1.84)	1.66 (1.43–1.93)	1.69 (1.44–1.99)	1.70 (1.43–2.02)	1.68 (1.40–2.01)
	80%	1.31 (1.22–1.40)	1.41 (1.29–1.55)	1.53 (1.37–1.72)	1.66 (1.45–1.90)	1.78 (1.52–2.09)	1.89 (1.58–2.26)	1.97 (1.61–2.41)	2.02 (1.62–2.51)	2.03 (1.61–2.55)	1.99 (1.56–2.53)
	0 mm	0.87 (0.84–0.89)	0.85 (0.81–0.88)	0.85 (0.80–0.89)	0.85 (0.80–0.91)	0.86 (0.80–0.93)	0.87 (0.80–0.95)	0.88 (0.80–0.97)	0.88 (0.79–0.98)	0.87 (0.78–0.98)	0.85 (0.76–0.96)
	5 mm	0.93 (0.92–0.94)	0.92 (0.90–0.94)	0.92 (0.90–0.94)	0.92 (0.89–0.95)	0.93 (0.89–0.96)	0.94 (0.90–0.98)	0.94 (0.89–0.99)	0.94 (0.89–0.99)	0.93 (0.88–0.99)	0.92 (0.87–0.98)
	10 mm	Ref.	Ref.	Ref.	Ref.	Ref.	Ref.	Ref.	Ref.	Ref.	Ref.
	15 mm	1.07 (1.06–1.09)	1.09 (1.06–1.11)	1.09 (1.06–1.12)	1.08 (1.05–1.12)	1.08 (1.04–1.12)	1.07 (1.02–1.12)	1.07 (1.01–1.12)	1.07 (1.01–1.12)	1.07 (1.01–1.13)	1.08 (1.02–1.15)
	20 mm	1.15 (1.12–1.19)	1.18 (1.13–1.23)	1.18 (1.12–1.25)	1.17 (1.10–1.25)	1.16 (1.07–1.25)	1.14 (1.05–1.25)	1.13 (1.03–1.25)	1.13 (1.02–1.26)	1.15 (1.02–1.28)	1.17 (1.04–1.32)
Total precipitation	25 mm	1.24 (1.19–1.29)	1.28 (1.21–1.36)	1.29 (1.19–1.39)	1.27 (1.16–1.40)	1.25 (1.11–1.40)	1.22 (1.07–1.39)	1.21 (1.04–1.40)	1.21 (1.03–1.42)	1.23 (1.03–1.46)	1.27 (1.06–1.52)
	30 mm	1.33 (1.26–1.40)	1.39 (1.28–1.51)	1.40 (1.26–1.55)	1.38 (1.21–1.56)	1.34 (1.15–1.56)	1.31 (1.10–1.56)	1.29 (1.06–1.56)	1.29 (1.04–1.59)	1.31 (1.05–1.65)	1.37 (1.08–1.74)
	35 mm	1.43 (1.33–1.53)	1.51 (1.37–1.67)	1.52 (1.34–1.74)	1.49 (1.27–1.75)	1.44 (1.20–1.74)	1.40 (1.13–1.74)	1.37 (1.07–1.75)	1.37 (1.05–1.79)	1.41 (1.06–1.87)	1.48 (1.10–2.00)
	40 mm	1.53 (1.41–1.67)	1.64 (1.45–1.85)	1.66 (1.42–1.94)	1.62 (1.34–1.96)	1.55 (1.24–1.95)	1.50 (1.15–1.94)	1.46 (1.09–1.96)	1.46 (1.06–2.01)	1.51 (1.07–2.12)	1.61 (1.12–2.30)
	45 mm	1.65 (1.49–1.81)	1.78 (1.55–2.05)	1.80 (1.50–2.16)	1.75 (1.40–2.19)	1.67 (1.28–2.18)	1.60 (1.18–2.17)	1.56 (1.11–2.19)	1.56 (1.07–2.26)	1.61 (1.08–2.40)	1.74 (1.14–2.65)
	50 mm	1.77 (1.58–1.97)	1.93 (1.65–2.27)	1.96 (1.59–2.42)	1.90 (1.47–2.45)	1.80 (1.33–2.43)	1.71 (1.21–2.42)	1.66 (1.12–2.45)	1.66 (1.08–2.54)	1.73 (1.10–2.72)	1.88 (1.17–3.04)

CI: Confidence Interval; IRR: Incidence Rate Ratio; LD: Legionnaires' Disease

IRRs and 95% CIs were estimated with a multivariable conditional quasi-Poisson model for community-acquired LD incidence at the municipality level. The model included three cross-bases, one for each area-weighted average climatic exposure (weekly mean temperature, weekly mean relative humidity and weekly total precipitation), with lags from 1 to 10 week. IRRs and 95% CIs are reported for individual week lags

Supplementary Figure S1. Number of community-acquired LD cases, by type of exposure and year of symptom

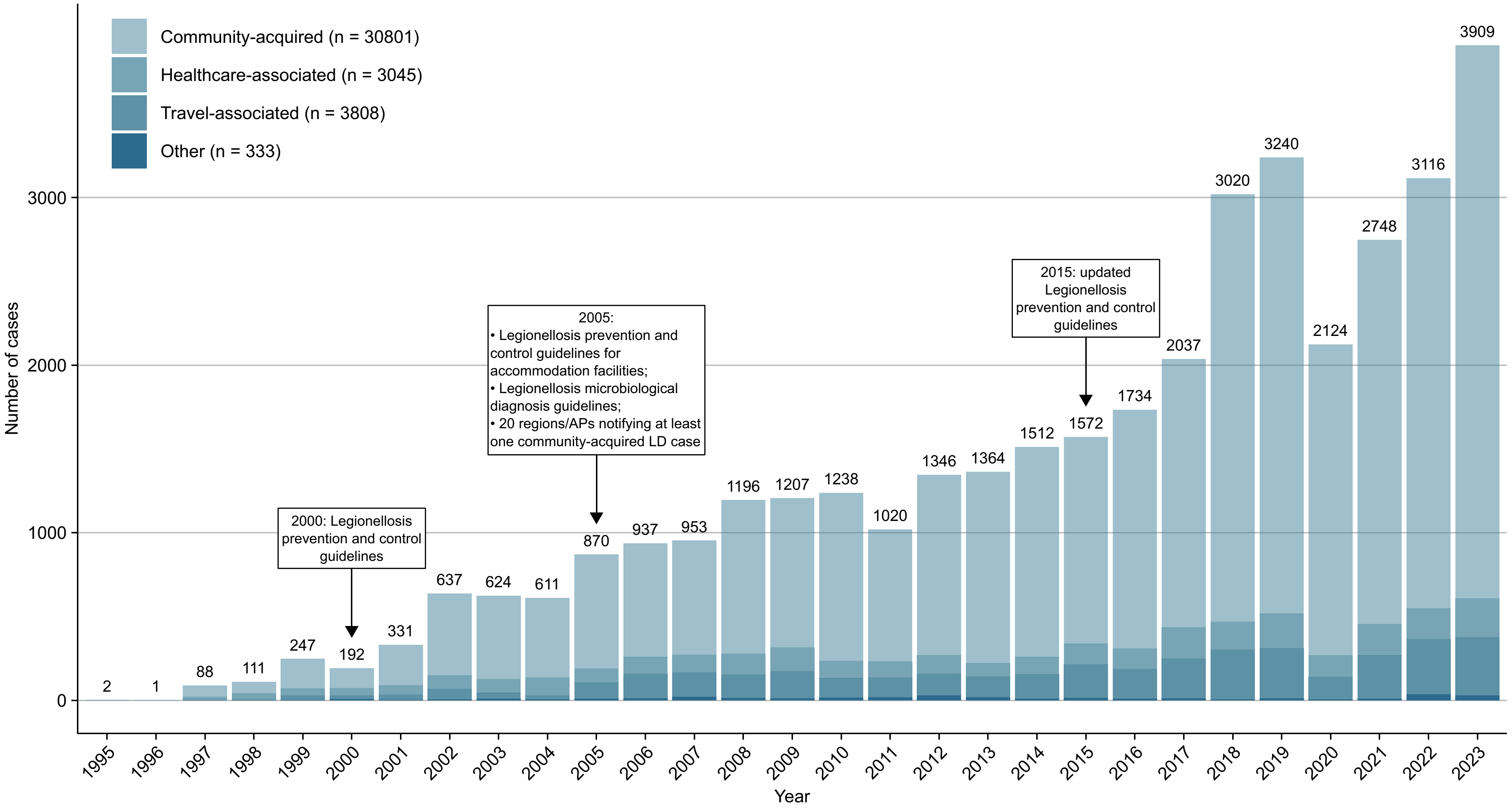

LD: Legionnaires' Disease

Supplementary Figure S2. Number of community-acquired LD cases (black curve, left axis) and temperature (panel A, red curve, right y-axis, °C), relative humidity (panel B, green curve, right y-axis, %) and total precipitation (panel C, blue curve, right y-axis, mm), by week of symptom onset

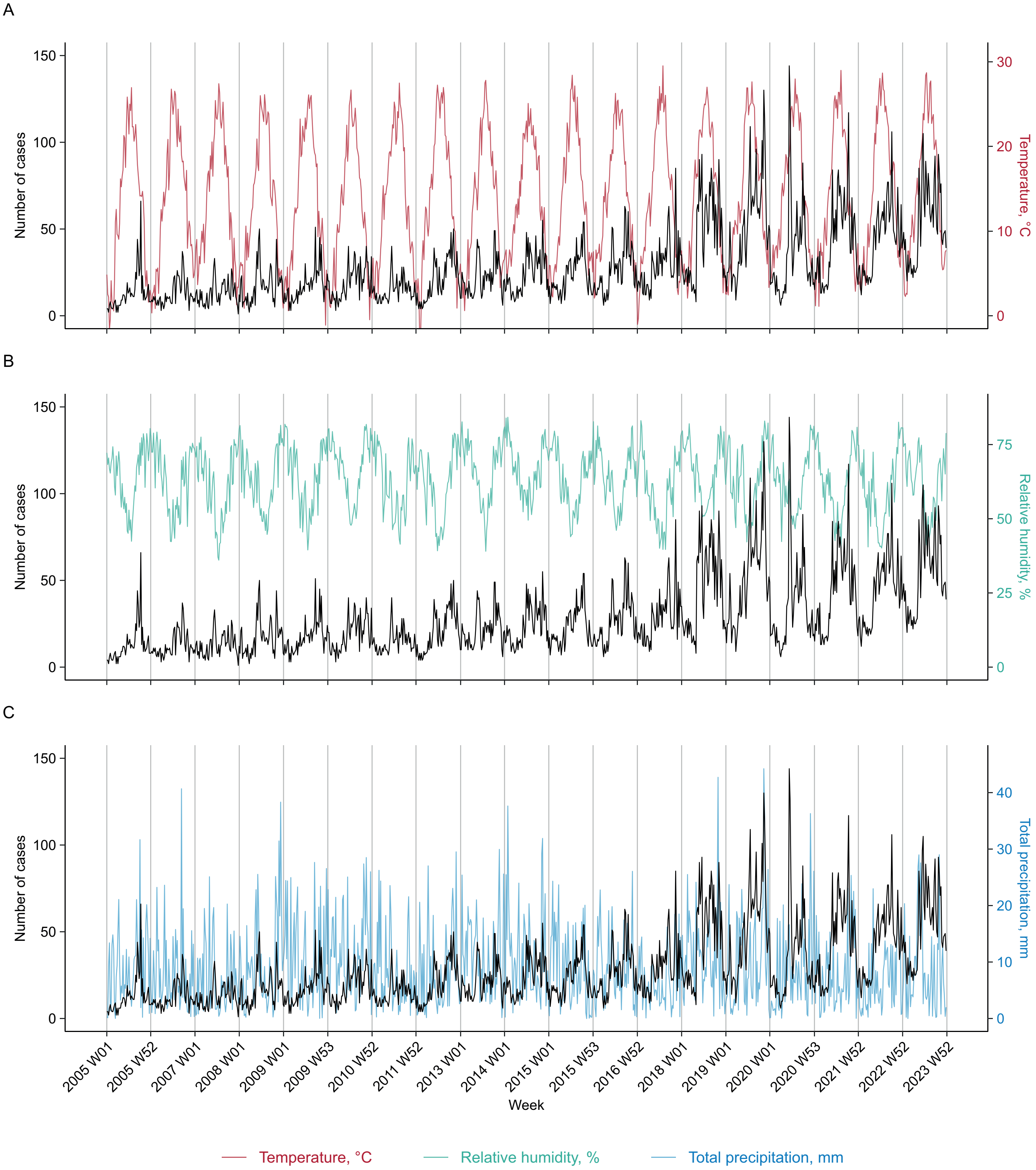

LD: Legionnaires' Disease

Supplementary Figure S3. Monthly proportion of community-acquired LD cases across years (panel A) and seasonality of community-acquired LD (panel B, IRR and 95% CI)

A

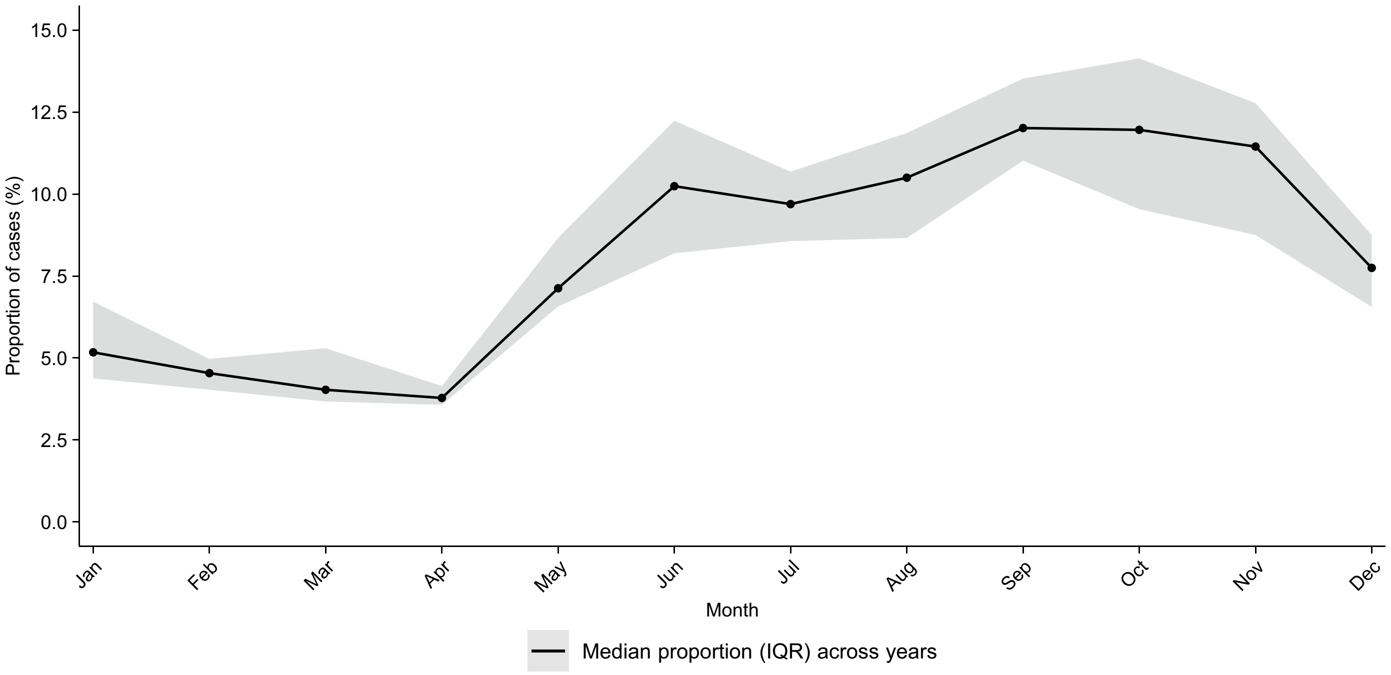

B

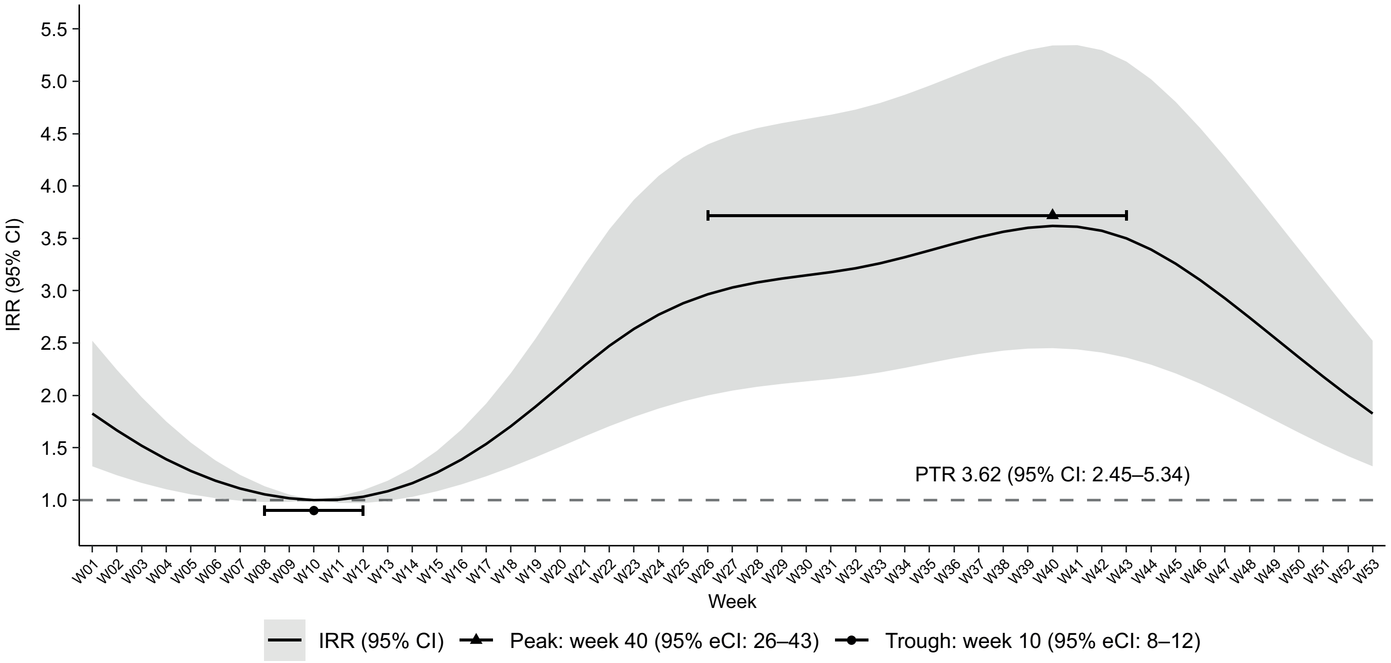

CI: Confidence Interval; eCI: empirical Confidence Interval; IRR: Incidence Rate Ratio; LD: Legionnaires' Disease; PTR: Peak-to-Trough Ratio

IRR is the ratio of LD incidence at each week-of-year to incidence at through week, and PTR is the ratio LD incidence at peak week to incidence at trough week

Supplementary Figure S4. Week lag-specific IRRs and 95% CIs for effect of temperature (panel A, ref. 15 °C), relative humidity (panel B, ref. 60%) and total precipitation (panel C, ref. 10 mm) on community-acquired LD incidence in population aged 60 or older at the municipality level (n. municipalities = 3326; n. community-acquired LD cases = 17,493)

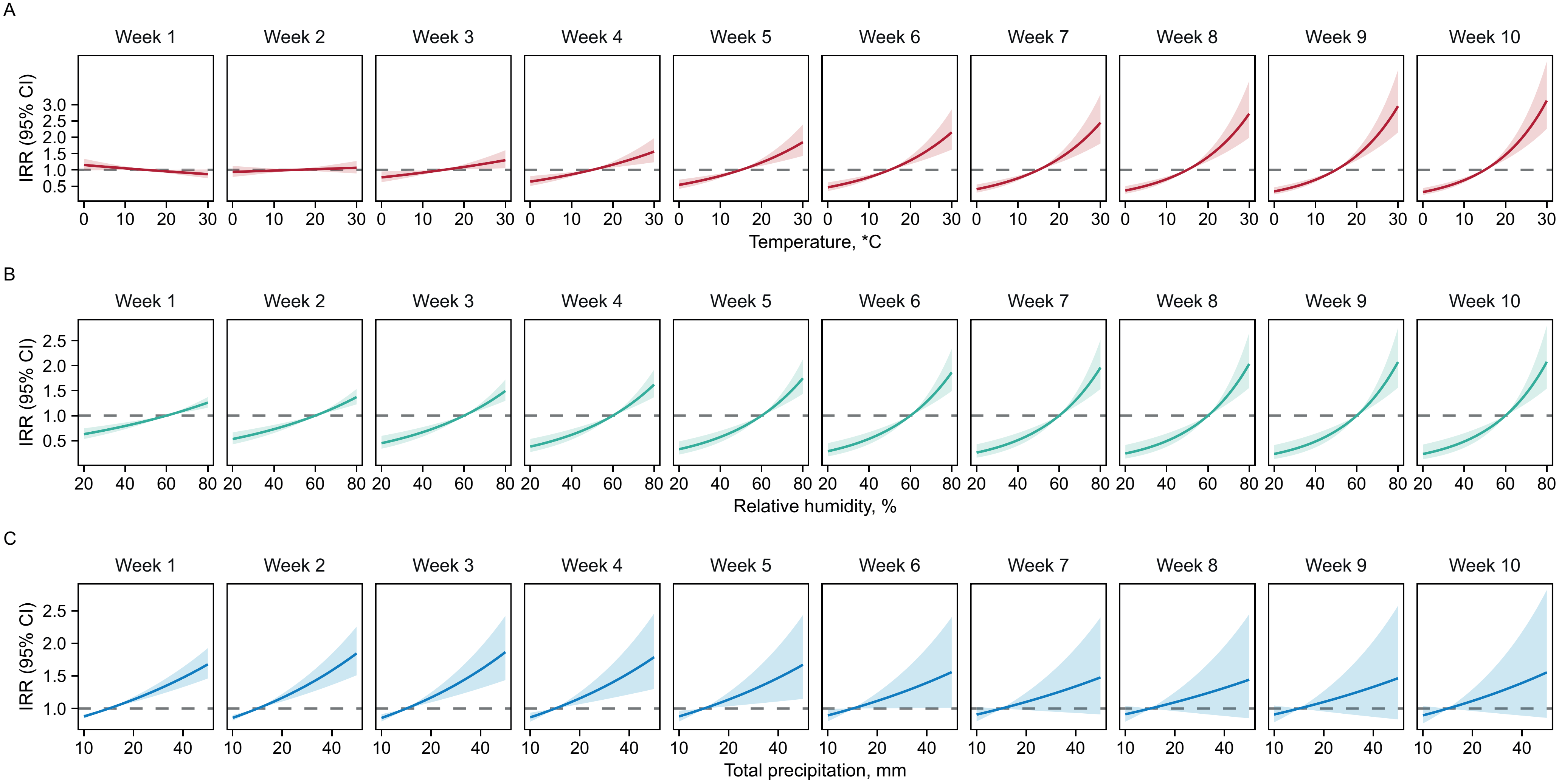

CI: Confidence Interval; IRR: Incidence Rate Ratio; LD: Legionnaires' Disease

IRRs and 95% CIs were estimated with a multivariable conditional quasi-Poisson model for community-acquired LD incidence at the municipality level. The model included three cross-bases, one for each area-weighted average climatic exposure (weekly mean temperature, weekly mean relative humidity and weekly total precipitation), with lags from 1 to 10 week. IRRs and 95% CIs are reported for individual week lags

Supplementary Figure S5. 1-, 5- and 10-week lag-specific temperature (ref. 15 °C), relative humidity (ref. 60%) and total precipitation (ref. 10 mm) combined IRRs, in population aged 60 or older at the municipality level (n. municipalities = 3326; n. community-acquired LD cases = 17,493)

		Temperature																	
Relative humidity	Total precipitation	5 °C	10 °C	15 °C	20 °C	25 °C	30 °C	5 °C	10 °C	15 °C	20 °C	25 °C	30 °C	5 °C	10 °C	15 °C	20 °C	25 °C	30 °C
20%	0 mm	0.61	0.58	0.55	0.53	0.50	0.48	0.19	0.23	0.29	0.35	0.43	0.53	0.10	0.14	0.21	0.30	0.44	0.65
	10 mm	0.69	0.66	0.63	0.60	0.57	0.55	0.22	0.27	0.33	0.40	0.49	0.60	0.11	0.16	0.23	0.34	0.50	0.72
	20 mm	0.79	0.75	0.72	0.68	0.65	0.62	0.25	0.30	0.37	0.46	0.56	0.69	0.12	0.18	0.26	0.38	0.55	0.81
	30 mm	0.90	0.85	0.82	0.78	0.74	0.71	0.28	0.34	0.42	0.52	0.64	0.78	0.14	0.20	0.29	0.42	0.62	0.90
	40 mm	1.02	0.97	0.93	0.89	0.85	0.81	0.32	0.39	0.48	0.59	0.72	0.89	0.15	0.22	0.32	0.47	0.69	1.01
	50 mm	1.16	1.11	1.06	1.01	0.96	0.92	0.36	0.44	0.55	0.67	0.82	1.01	0.17	0.25	0.36	0.53	0.77	1.13
40%	0 mm	0.77	0.73	0.70	0.67	0.63	0.61	0.33	0.41	0.50	0.62	0.76	0.93	0.20	0.29	0.43	0.63	0.92	1.35
	10 mm	0.87	0.83	0.79	0.76	0.72	0.69	0.38	0.47	0.57	0.70	0.86	1.06	0.23	0.33	0.48	0.70	1.03	1.50
	20 mm	0.99	0.95	0.90	0.86	0.82	0.78	0.43	0.53	0.65	0.80	0.98	1.20	0.25	0.37	0.54	0.79	1.15	1.68
	30 mm	1.13	1.08	1.03	0.98	0.94	0.89	0.49	0.60	0.74	0.91	1.11	1.37	0.28	0.41	0.60	0.88	1.28	1.88
	40 mm	1.28	1.23	1.17	1.12	1.07	1.02	0.56	0.68	0.84	1.03	1.27	1.56	0.31	0.46	0.67	0.98	1.43	2.10
	50 mm	1.46	1.40	1.33	1.27	1.21	1.16	0.63	0.78	0.96	1.17	1.44	1.77	0.35	0.51	0.75	1.09	1.60	2.34
60%	0 mm	0.96	0.92	0.88	0.84	0.80	0.76	0.58	0.72	0.88	1.08	1.33	1.63	0.42	0.61	0.90	1.31	1.91	2.80
	10 mm	1.10	1.05	1.00	0.95	0.91	0.87	0.66	0.81	1.00	1.23	1.51	1.85	0.47	0.68	1.00	1.46	2.14	3.13
	20 mm	1.25	1.19	1.14	1.09	1.04	0.99	0.75	0.93	1.14	1.40	1.71	2.10	0.52	0.76	1.12	1.63	2.39	3.49
	30 mm	1.42	1.36	1.30	1.24	1.18	1.13	0.86	1.05	1.29	1.59	1.95	2.39	0.58	0.85	1.25	1.82	2.67	3.90
	40 mm	1.62	1.55	1.47	1.41	1.34	1.28	0.98	1.20	1.47	1.81	2.22	2.72	0.65	0.95	1.39	2.04	2.98	4.35
	50 mm	1.84	1.76	1.68	1.60	1.53	1.46	1.11	1.36	1.67	2.05	2.52	3.10	0.73	1.06	1.56	2.27	3.32	4.86
80%	0 mm	1.22	1.16	1.11	1.06	1.01	0.96	1.02	1.25	1.54	1.89	2.32	2.85	0.87	1.27	1.86	2.72	3.98	5.82
	10 mm	1.38	1.32	1.26	1.20	1.15	1.10	1.16	1.43	1.75	2.15	2.64	3.24	0.97	1.42	2.08	3.04	4.44	6.50
	20 mm	1.58	1.50	1.43	1.37	1.31	1.25	1.32	1.62	1.99	2.44	3.00	3.68	1.09	1.59	2.32	3.39	4.96	7.25
	30 mm	1.79	1.71	1.63	1.56	1.49	1.42	1.50	1.84	2.26	2.78	3.41	4.19	1.21	1.77	2.59	3.79	5.54	8.10
	40 mm	2.04	1.95	1.86	1.77	1.69	1.62	1.71	2.10	2.57	3.16	3.88	4.76	1.35	1.98	2.89	4.23	6.19	9.05
	50 mm	2.32	2.22	2.12	2.02	1.93	1.84	1.94	2.38	2.93	3.59	4.41	5.42	1.51	2.21	3.23	4.72	6.91	10.10
Lag		Week 1						Week 5						Week 10					

IRR: 0.1 1.0 2.5 5.0 7.5 10.0 10.1

IRR: Incidence Rate Ratio; LD: Legionnaires' Disease

Combined IRRs were calculated by multiplying the exposure-specific IRRs estimated from the multivariable conditional quasi-Poisson model for community-acquired LD incidence at the municipality level. The model included three cross-bases, one for each area-weighted average climatic exposure (weekly mean temperature, weekly mean relative humidity and weekly total precipitation), with lags from 1 to 10 week. Combined IRRs are reported for individual week lags 1, 5 and 10
